# An Account of the Means Employed on Board His Majesty's Sloop Weasel to Preserve the Health of the Crew during a Voyage to Africa and the West Indies

**Published:** 1787

**Authors:** Leonard Gillespie

**Affiliations:** Surgeon in the Navy, and late Assistant Surgeon to His Majesty's Naval Hospital at St. Lucia


### LONDON MEDICAL JOURNAL,

FOR THE YEAR 1787,

### PART THE SECOND.

### I. *An Account of the Means employed on board His Majesty's Sloop Weasel to preserve the Health of the Crew during a Voyage to Africa and the West Indies; with a Reply to some Remarks lately published by M.* Chaussier, *Surgeon at Dijon. Communicated in a Letter to Dr.* Simmons *by Mr.* Leonard Gillespie, *Surgeon in the Navy, and late Assistant Surgeon to His Majesty's Naval Hospital at St. Lucia.*

HIS Majesty's sloop Weasel sailed from England for the coast of Africa in February, 1778, put into Teneriffe, where a quantity of wine was sent on board for the use of the ship's company, and from thence proceeding on her voyage, touched at Senegal, Gambia, Sierra Leona, Cape-coast Castle, and at the different factories on the slave coast as far down as Widah and the Gulph of Benin.

As the rainy season commenced soon after her arrival on the coast, her crew was inevitably bly expoſed to thoſe periodical rains ſo dangerous to European conſtitutions: to obviate their ill effects, a quantity of bark had been ſupplied to the ſurgeon previous to the ſhip's leaving England.

On rainy days, when the ſhip was at ſea, each ſeaman, on coming upon deck for his four hours watch, was ordered to ſtrip to his trowſers, in order to preſerve his frock, &c. dry, to put on when he ſhould come off deck; he had then a doſe of bark in wine adminiſtered to him, and the ſame was repeated when he was relieved; he then dipped himſelf in a tub of ſea water *, rubbed himſelf dry, put on his dry clothes, and went below.

When the ſhip was at anchor, and it was found neceſſary on rainy days to expoſe the people, by ſending them in boats, the ſame ſalutary diſcipline was obſerved.

Wine was ſerved in lieu of ſpirits as long as the ſtock laſted. The greateſt care was uſed to preſerve the births clean and well ventilated; ſcraping,

* The precaution of wetting with ſea water, after having been expoſed to the heavy rains in hot climates, is founded on the experience of the inhabitants of thoſe countries, who dread very much the ill effects of rain water externally applied,

ſcraping, waſhing with vinegar, and correcting the humidity between decks by means of fires, were practiſed. A ſick birth was formed under the forecaſtle *, and care was taken to keep the ſick ſeparated from thoſe in health.

By ſuch laudable care and attention on the part of the commander †, ſurgeon ‡, and other officers, this ſhip, with a complement of an hundred and twenty-five men, arrived in Engliſh Harbour, Antigua, in the month of October, without a ſick man on board, having had very few on her ſick liſt during the voyage, and having buried but one man ſince her departure from England. This, when compared with the ſtate of health enjoyed by Captain Cook's crew during his ſecond voyage, will plied, and have learnt to obviate them by immerſion in ſalt water.—Dr. Lind, in his Eſſay on the Diſeaſes of Europeans in hot Climates, recommends this practice. I have known ſpirits, externally applied, prevent the catching cold after being wet.

* The neceſſity of this regulation had been fatally experienced on board the Weaſel, on the ſame coaſt, in the year 1769, when an epidemic fever made its appearance two or three days after an infected perſon had been received on board.

† Captain Lewis Robinſon.

‡ Mr. William Telford.

 not not appear by any means extraordinary: but let us ſee the ſtate of health on board a ſhip on the ſame ſtation, in which the ſame ſalutary precautions were not obſerved, and we ſhall then be able to form a juſt eſtimate of the importance of means which may be uſed for preſerving the health of ſeamen on ſickly ſtations.

The Minerva, a frigate of thirty-two guns, ſailed from England for the coaſt of Africa a few days previous to the ſailing of the Weaſel: unfortunately, her allowance of bark had not been ſent on board, but was put on board the Weaſel, in order to be conveyed to her when the ſhips ſhould meet on the coaſt; but that did not happen, and of courſe there was no bark iſſued to the Minerva's ſhip's company. What precautions were uſed, or neglected, on board the Minerva, I am unacquainted with; but it is certain that when ſhe arrived at Sierra Leona, the third or fourth ſtation for men of war in running down the coaſt, a great proportion of her ſhip's company was ill of fever; and the commander, who was himſelf affected with the reigning epidemic, the conſequences of which proved fatal to him, convinced of the danger to thoſe under his command, of perſiſting to remain on the coaſt with a ſickly

ſhip's

ſhip's company, bore away for the Weſt Indies; where, after burying a number of men, this frigate arrived in a condition ſo wretched, that ſhe fell an eaſy prize to an enemy's frigate.

I cannot finiſh this letter without taking notice of ſome remarks made by Monſieur Chauſſier, Surgeon at Dijon, in a letter of his publiſhed in Mr. Lombard's third volume of *Opuſcules de Chirurgie*, (page 346) printed at Straſbourg in 1786, and which you were ſo polite as to favour me with a ſight of, on a note inſerted in my paper on the putrid ulcer, publiſhed in the London Medical Journal for 1785. The note alluded to is that* in which I ſay I am convinced many men fall a ſacrifice to the uſe of large inciſions in the Hotel Dieu at Paris. In making this aſſertion, I have only advanced a fact, unwillingly drawn from me, through a regard to the intereſts of humanity. Were I inclined to hurt the feelings of individuals, I might eaſily ſupport my aſſertion by publiſhing an account of ſome caſes, but that would be invidious; and it would alſo be unneceſſary, as the Hotel Dieu is now under the care of a gentleman†,

* Vol. VI. page 387.

† Monſ. Deſault.

who, who, to great experience and fcience, joins the greateft humanity, and the moft affiduous application in the difcharge of the important charge entrufted to him. Had I advanced any thing untrue, a refutation of it would have come with more force from Paris than from Dijon or Strafbourg. But what feems to have given Meffrs. Chauffier and Lombard moft offence in the note above alluded to, is the imputation feemingly thrown out on the French furgeons in general, that they are too fond of large incifions, and are not in the practice of ufing the feton.—Although a foreigner in Paris may perhaps be juftifiable in fuppofing that the practice which he fees in the hofpitals, and the doctrine he hears inculcated in the fchools, form the practice of the moft enlightened furgeons of the nation; yet I confefs that I was wrong in too haftily throwing out a reflection in the leaft injurious to a body fo liberal and fo enlightened as the French furgeons. The improvements which furgery has received from Paré, Mauriceau, La Motte, Le Dran, Petit, and a number of other excellent French furgeons, and the liberal eftablifhments which the munificence of the French monarchs have induced them to keep open for ftudents of all nations, nations, have perhaps tended more to improve ſurgery, during the two laſt and preſent centuries, than the joint labours of the ſurgeons of one half of Europe beſides.—The inſcription on the noble amphitheatre in the College of Surgery at Paris, erected during the late reign, conveys a juſt eulogium on that humane, polite, and liberal nation—

"Ad cædes hominum priſca amphitheatra patebant;

"Ut longum diſcant vivere noſtra patent."

*Lothbury,*
March 4th, 1787.

